# Skating

**Published:** 1860-02

**Authors:** 


### SKATING

Is one of the manliest and most invigorating of all forms of exercise, and it is due to the Commissioners of the Central Park, to say that the pains taken by them to afford to our citizens the facilities of skating have met with public appreciation. If the ice did not freeze smooth, it was overflowed and frozen over again. If the snow fell upon it, it was promptly removed; and when the ice became cut up, it was again overflowed, and thus during the cold weather, thousands and tens of thousands of men, women, and children have found innocent, exciting, and healthy amusement. A separate lake of ice was prepared for the ladies, with an apartment on the bank for convenience of dressing, resting, warming, etc. On several occasions, especially on the day celebrated for New Year's, we were one of the skaters, and among the thousands on the ice at the same time, we did not hear an oath, an unbecoming, or even angry word.

